# Molecular Markers of Tubulointerstitial Fibrosis and Tubular Cell Damage in Patients with Chronic Kidney Disease

**DOI:** 10.1371/journal.pone.0136994

**Published:** 2015-08-28

**Authors:** Shunsaku Nakagawa, Kumiko Nishihara, Hitomi Miyata, Haruka Shinke, Eri Tomita, Moto Kajiwara, Takeshi Matsubara, Noriyuki Iehara, Yoshinobu Igarashi, Hiroshi Yamada, Atsushi Fukatsu, Motoko Yanagita, Kazuo Matsubara, Satohiro Masuda

**Affiliations:** 1 Department of Clinical Pharmacology and Therapeutics, Kyoto University Hospital, Sakyo-ku, Kyoto, 606–8507, Japan; 2 Department of Nephrology, Graduate School of Medicine, Kyoto University, Sakyo-ku, Kyoto, 606–8507, Japan; 3 Toxicogenomics Informatics Project, National Institutes of Biomedical, Innovation, Health and Nutrition, 7-6-8, Saito-Asagi, Ibaraki, Osaka, 567–0085, Japan; Tokushima University Graduate School, JAPAN

## Abstract

In chronic kidney disease (CKD), progressive nephron loss causes glomerular sclerosis, as well as tubulointerstitial fibrosis and progressive tubular injury. In this study, we aimed to identify molecular changes that reflected the histopathological progression of renal tubulointerstitial fibrosis and tubular cell damage. A discovery set of renal biopsies were obtained from 48 patients with histopathologically confirmed CKD, and gene expression profiles were determined by microarray analysis. The results indicated that hepatitis A virus cellular receptor 1 (also known as Kidney Injury Molecule-1, KIM-1), lipocalin 2 (also known as neutrophil gelatinase-associated lipocalin, NGAL), SRY-box 9, WAP four-disulfide core domain 2, and NK6 homeobox 2 were differentially expressed in CKD. Their expression levels correlated with the extent of tubulointerstitial fibrosis and tubular cell injury, determined by histopathological examination. The expression of these 5 genes was also increased as kidney damage progressed in a rodent unilateral ureteral obstruction model of CKD. We calculated a molecular score using the microarray gene expression profiles of the biopsy specimens. The composite area under the receiver operating characteristics curve plotted using this molecular score showed a high accuracy for diagnosing tubulointerstitial fibrosis and tubular cell damage. The robust sensitivity of this score was confirmed in a validation set of 5 individuals with CKD. These findings identified novel molecular markers with the potential to contribute to the detection of tubular cell damage and tubulointerstitial fibrosis in the kidney.

## Introduction

Physiological changes in the course of chronic kidney disease (CKD) produce a complex series of outcomes, including loss of kidney function, leading to end-stage renal failure. In CKD, the irreversible and progressive loss of nephrons results in glomerular sclerosis, tubular atrophy, tubulointerstitial fibrosis, and eventually further reductions in nephron numbers. Pathological changes such as tubular cell atrophy and interstitial fibrosis are commonly seen in progressive renal disease, irrespective of the initial etiology; these are cardinal features of CKD [1, 2]. However, the molecular characteristics of these pathological events remain to be fully understood.

Microarray analysis facilitates a genome-wide survey of mRNA transcripts; this approach can be used to study the active biological processes in animal models of renal injury [3, 4] and in human kidney disease [5, 6]. Halloran et al. adapted microarray analysis for renal transplant biopsies, revealing gene expression differences associated with future graft loss or acute kidney injury in renal transplant patients [7, 8]. In addition, renal biopsy microarray data was useful for the prediction of graft survival [9]. Naesens et al. used microarrays to profile renal allografts and identified a molecular signature associated with tubular atrophy and interstitial fibrosis following kidney transplantation [10]. These findings suggested that transcriptional profiling of renal biopsy specimens from CKD patients could assist with diagnosis or therapeutic intervention in this condition. Furthermore, they provided information that may contribute to the identification of useful CKD biomarkers in urine and other body fluids.

We conducted a microarray analysis of renal biopsy specimens from CKD patients in order to identify gene expression differences that associated with the histopathological grade of the biopsies. The studies using a rodent CKD model validated a novel gene set and related this to tubulointerstitial fibrosis and tubular cell injury. In addition, we developed a molecular score using the microarray profiles, and assessed its performance and usefulness for the classification of the grade of tubulointerstitial fibrosis and tubular cell damage.

## Materials and Methods

### Patients and Biopsies

This study was conducted in accordance with the Declaration of Helsinki and its amendments and was approved by Kyoto University Graduate School and the Faculty of Medicine Ethics Committee. All patients gave their written informed consent. A total of 48 patients with histopathologically confirmed CKD were enrolled into the study as a discovery set. Kidney biopsies were obtained between October 2008 and October 2010. The characteristics of these patients are summarized in Table 1. The patients had various renal diseases that were histologically confirmed as IgA nephropathy (n = 15), membranous nephropathy (n = 7), lupus nephritis (n = 6), minimal change nephrotic syndrome (n = 3), membranoproliferative glomerulonephritis (n = 3), amyloidosis (n = 3), antineutrophil cytoplasmic antibody- related glomerulonephropathy (n = 2), diabetic nephropathy (n = 2), and other nephropathies (n = 6).

**Table 1:** Characteristics of Patients in the Discovery Set.

Variables	Discovery set (n = 48)
Age (y)	48.3 ± 18.7
Gender (male/female, n)	24 / 24
Serum creatinine (mg/dl)	0.96 ± 0.41
Blood urea nitrogen (mg/dl)	16.7 ± 8.4
**Primary disease (n)**	
IgA nephropathy	15
Membranous nephropathy	7
Minimal change nephrotic syndrome	4
Membranoproliferative glomerulonephropathy	3
Diabetic nephropathy	2
Lupus nephritis	6
Amyloidosis	3
ANCA-related glomerulonephropathy	2
Others	6
**Histopathology (grade 0–4)**	
Tubular cell damage grade	1.9 ± 1.1 (1–4)
Tbulointerstitial fibrosis grade	2.1 ± 1.0 (1-4)

Values are n or mean ± standard deviation.

ANCA, antineutrophil cytoplasmic antibody.

In addition to the discovery set, 5 patients were enrolled to a validation set, and kidney biopsies were obtained between May 2012 and June 2012 (Table 2).

**Table 2:** Characteristics of Patients in the Validation Set.

Subject	Age (y)	Gender	Serum creatinine (mg/dl)	Blood urea nitrogen (mg/dl)	Tubular cell damage grade (0–5)	Tubulointerstitial fibrosis grade (0–5)	Primary disease
A	70	Male	9.8	103	4	3	Rapid progressive glomerulonephritis
B	64	Male	0.6	23	1	1	Membranous nephropathy
C	70	Male	0.9	18	2	2	IgA nephropathy
D	26	Female	2.7	32	4	4	Diabetic nephropathy
E	27	Male	1.6	11	5	3	Chronic tubulointerstitial nephritis

All biopsies were assessed by pathologists who were blinded to the results of the microarray studies. Tubular cell damage and tubulointerstitial fibrosis were evaluated and a severity scale of 0–5 was used to grade the pathological lesions observed. The scaling was defined according to the area of tubular lesion or fibrosis: grade 0, 0%; grade 1, up to 10%; grade 2, 10–30%; grade 3, 30–50%; grade 4, more than 50%; grade 5, almost 100%.

### RNA Extraction and Microarrays

To stabilize and maintain the integrity of tissue RNA, the obtained biopsy specimens were placed in RNA*later* (Life Technologies Corp., Carlsbad, CA, USA) according to the manufacturer’s instruction, and stored at -80°C until further use. Total RNA was isolated from biopsy specimens using an RNeasy micro Kit (Qiagen, Hilden, Germany) according to the manufacturer’s instructions, and the concentrations of total RNA were measured by spectrophotometry. The quality of total RNA was investigated using a 2100 Bioanalyzer (Agilent Technologies, Santa Clara, CA). Cyanine-3-labeled complementary (c) RNA was generated using a Quick Amp Labeling Kit (Agilent Technologies). Array hybridization, fluorescence detection, and image acquisition were performed in accordance with the manufacturer’s instructions. In brief, 1.0 μg of each cRNA sample was hybridized to a Whole Human Genome Microarrays (4 × 44K G4112F, Agilent Technologies) at 65°C for 17 h. After the arrays were washed, fluorescent signals were detected using a DNA Microarray Scanner (Agilent Technologies).

### Microarray Data Analysis

The results of the microarray assays were analyzed using Feature Extraction Software (Agilent Technologies). The raw data sets for the 53 biopsies included in the present study have been deposited at the Gene Expression Omnibus under GSE (accession number: GSE66494). Bad spots were flagged prior to normalizing the assay signals across the experiments with each median. Human adult normal kidney total RNA, purchased from BioChain (Newark, CA), was used as the control. Detectable signals were selected if the mean signal of a feature was greater than that of the corresponding background and if this difference was statistically significant.

The ratio of the mean signals from the 48 biopsies in the validation set to those of the control kidney RNA was calculated, and genes with a z-score > 2.0 or < -2.0 were considered to be up- or down-regulated, respectively. These genes were classified according to their function using MetaCore Software (GeneGo, St. Joseph, MI). Furthermore, the microarray signals for these genes were compared between groups using the Kruskall-Wallis test. Differences were considered to be statistically significant if the probability value (P) was < 0.05. A molecular score was calculated using the geometric mean of the fold change (versus control kidney RNA) of the values for 5 genes; hepatitis A virus cellular receptor 1 (HAVCR1; also known as Kidney Injury Molecule-1, KIM-1), lipocalin 2 (LCN2; also known as neutrophil gelatinase-associated lipocalin, NGAL), SRY-box 9 (SOX9), WAP four-disulfide core domain 2 (WFDC2), and NK6 homeobox 2 (NKX6-2). This calculation was performed using an adaptation of a previously published method [10]. The area under the receiver operating characteristics curve (AUC-ROC) was calculated and used to assess the performance of this molecular score. Statistical analysis was performed using Prism Version 4.0 software (Graphpad, San Diego, CA).

### Animals

A total of 26 C57BL/6 male mice (6-week-old) were purchased from SLC Animal Research Laboratories (Shizuoka, Japan), housed under specific pathogen free conditions at the animal care facility at the Guidelines for Animal Experiments of Kyoto University. All protocols were approved by the Animal Research Committee, Graduate School of Medicine, Kyoto University. The mice were kept in a temperature-controlled (25 ± 2 degree Centigrade) environment with a 12-h light/dark cycle and received a standard diet and water ad libitum. Every efforts made to minimize the number and suffering of animals used. Complete unilateral ureteral obstruction (UUO) was performed as previously described [11]. Under sodium pentobarbital anesthesia, the middle portion of the left ureter was ligated in 2 places and cut between these points. The mice were sacrificed 7 or 14 days after surgery and their kidneys were subjected to subsequent investigations to examine the gene expression profiles after injury (7 mice in each group). Sham-operated rats were used as controls at the same time-points as the rats with UUO (6 mice in each group).

### Real-Time PCR

Whole mouse kidney total RNA was extracted with an RNeasy Mini Kit (Qiagen). Total RNA was reverse-transcribed with random hexamers using Superscript II reverse transcriptase (Invitrogen, Life Technologies Corp.) and digested with RNase H (Invitrogen). Real-time PCR was carried out using the Power SYBR Green PCR Master Mix (Applied Biosystems) and an Applied Biosystems StepOnePlus Real-Time PCR System (Applied Biosystems), according to manufacturer’s instructions. The following primers were used: CAGGCATGGATGGCATCAATCAC and ACTCTAGCTGTGAAGTCAGTGTCG for *Acta2*; TTCAAGTCTTCATTTCAGGCC and CTCTGATGTGTGACCGTATAT for *Havcr1*; CTCAGAACTTGATCCCTGCC and TCCTTGAGGCCCAGAGACTT for *Lcn2*; AACCAATTACGGACTGTGTGTT and TCGCTCGGTCCATTAGGCT for *Wfdc2*; CGGAGGAAGTCGGTGAAGA and GTCGGTTTTGGGAGTGGTG for *Sox9*; and AAGTCTGCCCCGTCTCAAC and GGTCTGCTCGAAAGTCTTCTC for *Nkx6-2*. Glyceraldehyde-3-phosphate dehydrogenase (*GAPDH*) mRNA was also measured as an internal control using TaqMan Rodent GAPDH Control Reagent (Applied Biosystems). Data were expressed as mean ± standard deviation of the mean (SD). The data were analyzed statistically by one-way analysis of variance (ANOVA) to account for multiple testing. P values < 0.05 were considered statistically significant. Statistical analysis was performed using Prism Version 4.0 software (Graphpad).

### Western blotting

Urine samples collected from the ureters of mice were separated using sodium dodecyl sulfate polyacrylamide gel electrophoresis and blotted onto polyvinylidene difluoride membranes (Merck Millipore, Darmstadt, Germany). Membranes were blocked, washed, and incubated overnight at 4°C with primary antibodies to mouse Kim-1 (Abcam, Cambridge, UK) and mouse Ngal (Santa Cruz Biotechnology, Avenue, CA, USA). The bound antibodies were detected on X-ray film by using enhanced chemiluminescence with horseradish peroxidase (HRP)-conjugated secondary antibodies and cyclic diacylhydrazides (Merck Millipore).

## Results

### Gene Expression Profiles in Renal Biopsies from CKD Patients

**Fig 1:**
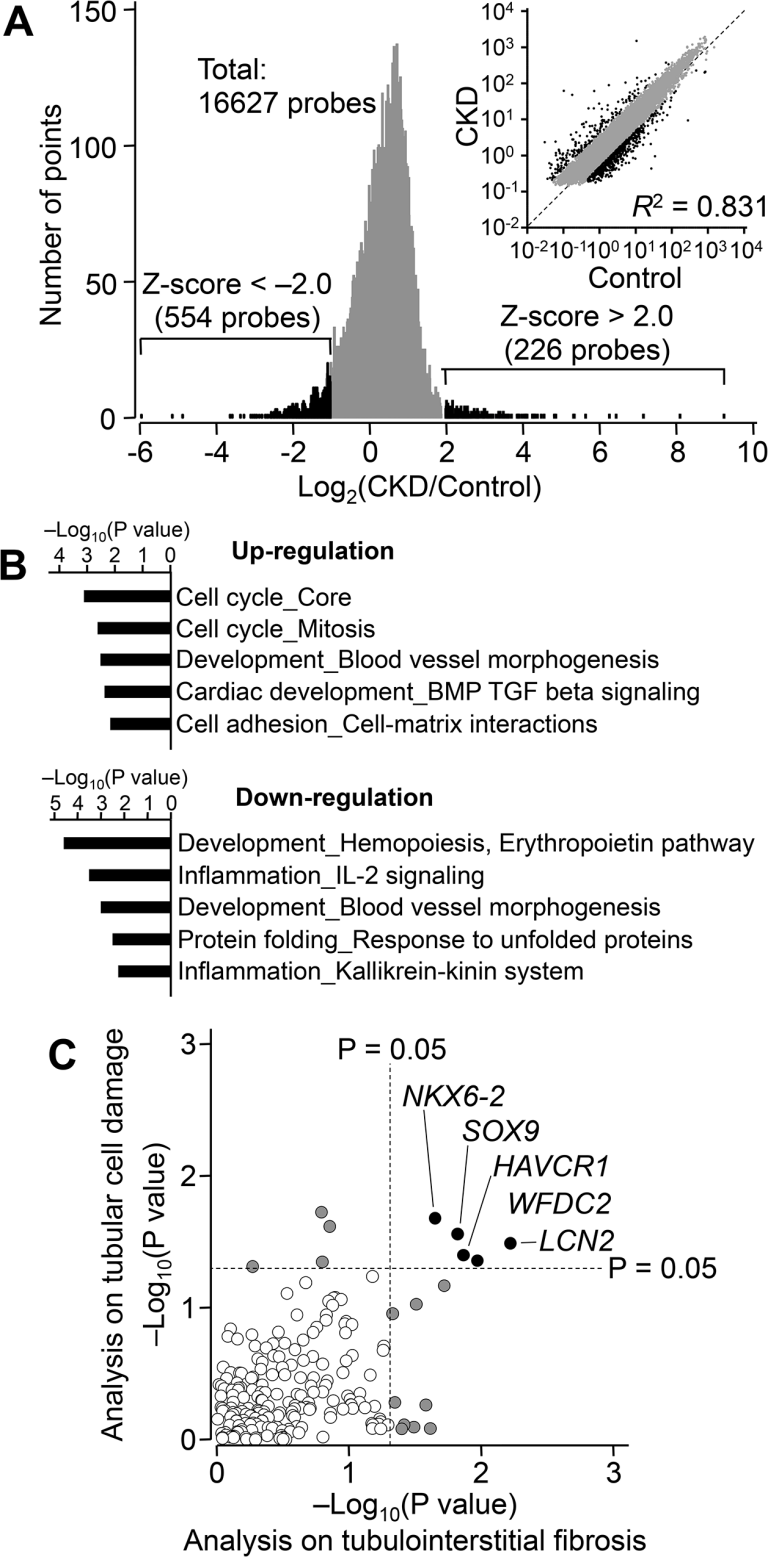
The Transcriptional Profile Related to Chronic Kidney Disease (CKD). (**A**) Distribution of differences in gene expression between control kidney RNA (Control) and RNA extracted from 48 CKD biopsies. Microarray analysis was performed and genes down-regulated (z-score < -2.0, green) or up-regulated (z-score > 2.0, blue) in CKD are indicated. (**B**) Biological functions of the genes showing differential expression in CKD samples were classified according to their Gene Ontology and P values were calculated with MetaCore software. (**C**) Relationship between P values from Kruskal-Wallis test on tubulointerstitial fibrosis and tubular cell damage. Each symbol represents one gene. Gray or black circles indicate genes with any P values < 0.05; open circles represent genes with both P values > 0.05. HAVCR1, hepatitis A virus cellular receptor 1; LCN2, lipocalin 2; SOX9, SRY-box 9; WFDC2, WAP four-disulfide core domain 2; NKX6-2, NK6 homeobox 2.

To characterize transcriptional profiles in CKD, we analyzed a discovery set of renal biopsies from 48 different CKD patients (Table 1). First, the differences between the gene expression profiles of these 48 biopsies and that of a control kidney RNA sample were assessed by microarray analysis. Using a z-score cut-off of ± 2.0, we identified 554 down-regulated and 226 up-regulated genes (Fig 1A). We classified these by Gene Ontology using MetaCore software in order to identify key biological functions involved in CKD (Fig 1B). This analysis indicated that genes related to cell cycle progression were significantly increased in renal biopsies of CKD patients. Additionally, levels of transcripts in the erythropoietin pathway and interleukin-2 signaling were decreased in these CKD samples.

We next searched within the 226 up-regulated genes for molecular changes related to tubulointerstitial fibrosis and tubular cell damage. To identify genes associated with kidney pathophysiology, we focused on the transcriptional profiles of patients with primary glomerulonephritis and diabetic nephropathy. Thirty-one patients diagnosed with IgA nephropathy, membranous glomerulonephropathy, minimal change mesangial proliferative glomerulonephropathy, membranoproliferative glomerulonephritis, or diabetic nephropathy were selected for further analysis. The relationship between transcriptional changes and the histopathology grade of each sample was analyzed using the Kruskall-Wallis test. We identified 14 genes showing a significant correlation with tubulointerstitial fibrosis and 9 genes showing a significant correlation with tubular cell damage (P < 0.05; Fig 1C, gray or black circles). Notably, HAVCR1 (KIM-1), LCN2 (NGAL), SOX9, WFDC2, and NKX6-2 showed a significant relationship with both tubulointerstitial fibrosis and tubular cell damage (Fig 1C, black circles). We also confirmed that the selection of patient did not significantly affect the results obtained in this analysis (S1 Fig).

### Gene Expression Profiles in a Mouse UUO model

**Fig 2:**
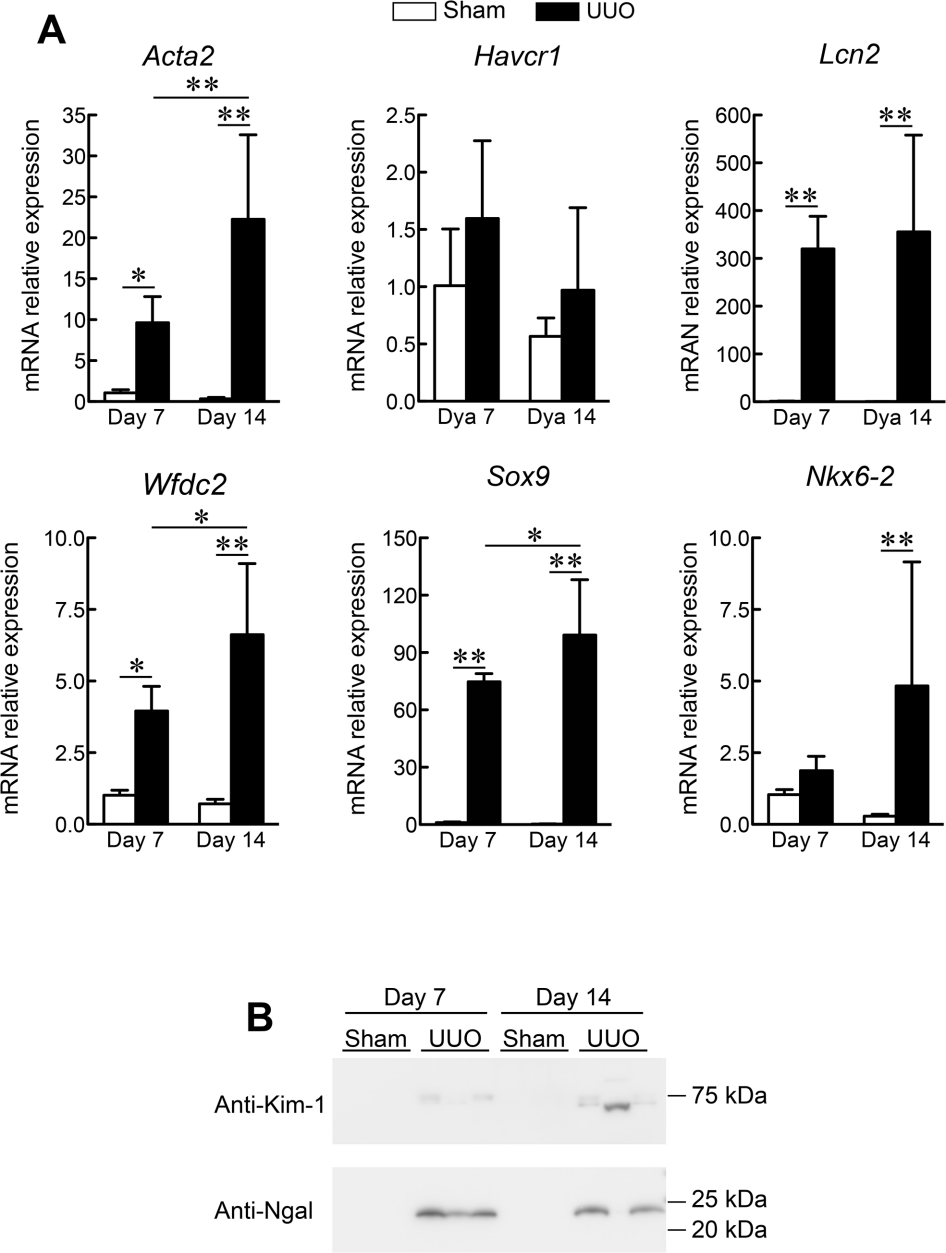
Mouse Kidney Gene Expression Profiles following Unilateral Ureteral Obstruction (UUO). (A) Real-time PCR analysis was used to determine the relative mRNA levels of alpha smooth muscle actin (Acta2), hepatitis A virus cellular receptor 1 (Havcr1), lipocalin 2 (Lcn2), SRY-box 9 (Sox9), WAP four-disulfide core domain 2 (Wfdc2), and NK6 homeobox 2 (Nkx6-2) at the indicated time-points. Glyceraldehyde-3-phosphate dehydrogenase *(GAPDH)* mRNA was used as an internal control. Data are expressed as the mean ± standard deviation of 4–6 mice. *P < 0.05; **P < 0.01; one-way analysis of variance. (B) Western blot analysis was used to determine the leakage of Ngal (Lcn2) and Kim-1 (Havcr1) in mouse urine.

To explore whether the mRNA expression levels of the 5 selected genes reflected tubulointerstitial fibrosis or tubular cell damage, we used a rodent UUO model of renal fibrosis (Fig 2A). Seven and 14 days after surgery, the mRNA expression level of alpha smooth muscle actin (Acta2) was strongly up-regulated in the mouse kidneys, indicating progressive interstitial fibrosis. We found significant up-regulation of Lcn2, Wfdc2, Sox9 and Nkx6-2 in these kidneys. Notably, the expression levels of Wfdc2 and Sox9 were increased as the kidney damage progressed. The expression of Havcr1 also tended to increase in tandem with kidney damage, although this relationship was not statistically significant. On the other hand, the leakage of Ngal (Lcn2) and Kim-1 (Havcr1) proteins in the urine were found after UUO (Fig 2B). These results of the rodent UUO kidney injury model validated those obtained by microarray analysis of human renal biopsies.

### Defining Gene Set Scores as Quantitative Parameters for Progressive Tubular Damage

**Fig 3:**
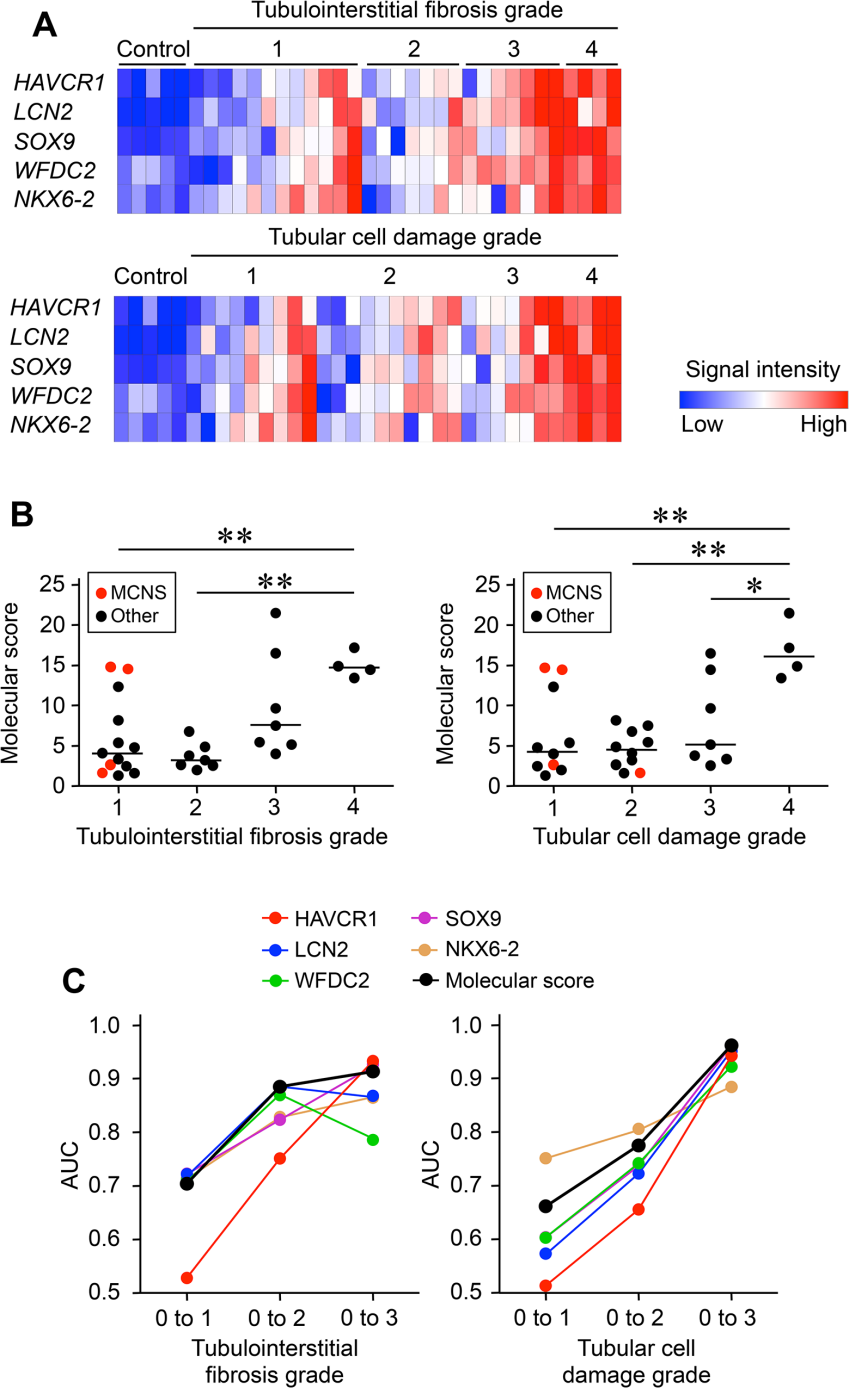
Analysis of the Molecular Score Performance. (**A**) Relative signal intensity heatmaps for hepatitis A virus cellular receptor 1 (HAVCR1), lipocalin 2 (LCN2), SRY-box 9 (SOX9), WAP four-disulfide core domain 2 (WFDC2), and NK6 homeobox 2 (NKX6-2) in relation to renal histopathology. (**B**) The distribution of molecular score based on histological grades. (**C**) The area under the receiver operating characteristics curve (AUC) for the molecular score of each biopsy, plotted against the grade of tubulointerstitial fibrosis and tubular cell damage.

The microarray data provided evidence of an evolution in the expression of HAVCR1, LCN2, SOX9, WFDC2, and NKX6-2 that mirrored the histological progression of tubulointerstitial fibrosis and tubular cell damage (Fig 3A). We therefore explored the usefulness of HAVCR1, NGAL, SOX9, WFDC2, and NKX6-2 as biomarkers for these renal histopathological changes. To develop a gene-based classifier of the severity of tubulointerstitial fibrosis and tubular cell damage, we built a molecular score based on the expression of these 5 genes, as compared with control kidney RNA. This score was calculated as the geometric mean of the fold change (unlogged values) across all probe sets, by adapting a previously published method [10] (Fig 3B). The scores increased with the severity of histopathological changes. In cases with minimal change nephrotic syndrome, 2 patients showed high molecular scores while the histological grades were low. The sensitivity and specificity of this score for histological changes was then evaluated by composite AUC-ROC analysis (Fig 3C). Although the molecular score performed poorly for low-grade histopathology (low AUC values for grade 1 versus grades 2–4), the AUC-ROC analysis showed that it performed well in the discrimination of grades 1–2 versus grades 3–4, and of grades 1–3 versus grade 4.

### Molecular Score-Based Prediction of the Severity of Tubular Damage

**Fig 4:**
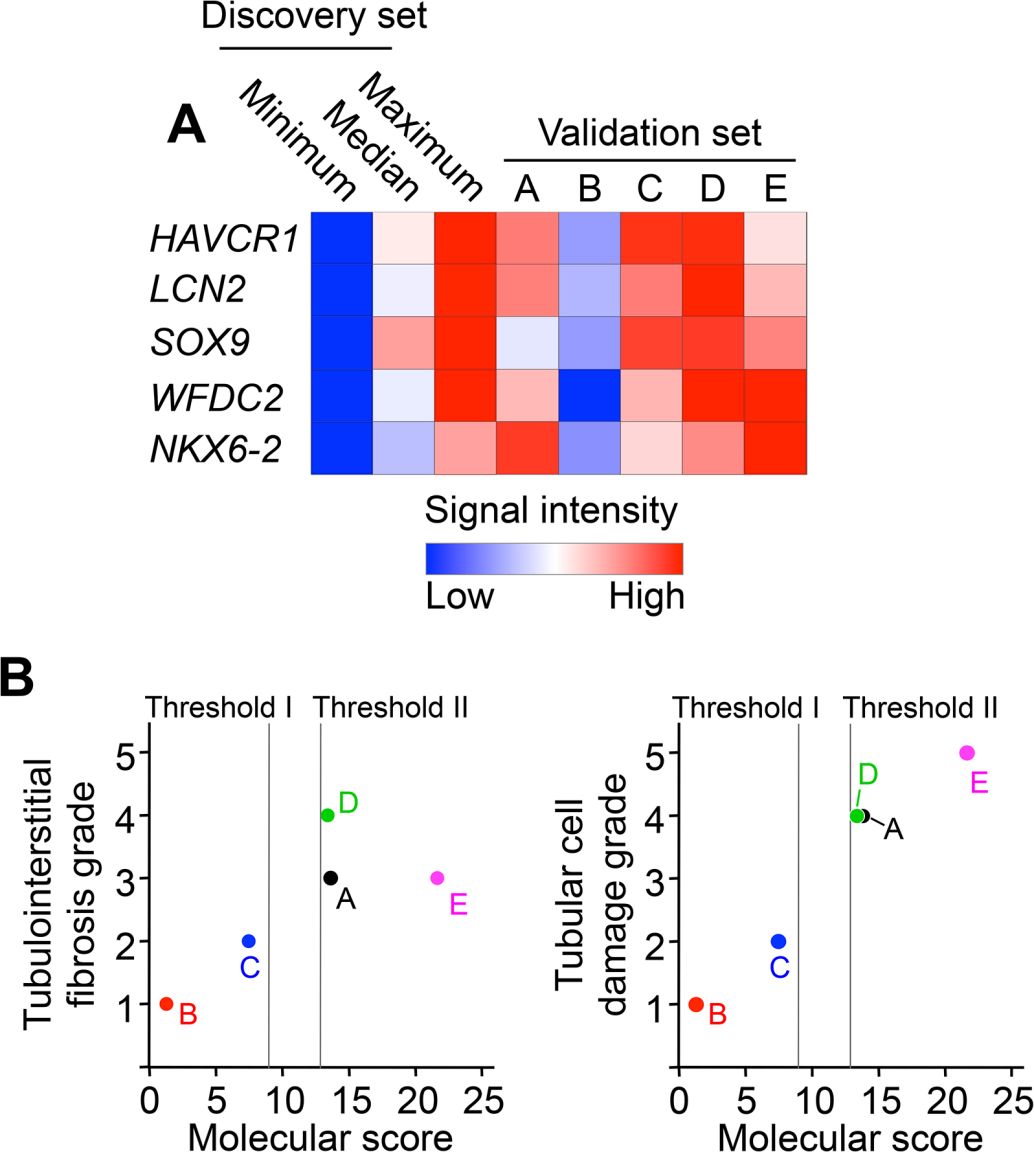
Validation Set Study. Microarray analysis was performed on 5 biopsies from CKD patients in the validation set. (**A**) Heatmap of the relative signal intensities of hepatitis A virus cellular receptor 1 (HAVCR1), lipocalin 2 (LCN2), SRY-box 9 (SOX9), WAP four-disulfide core domain 2 (WFDC2), and NK6 homeobox 2 (NKX6-2). (**B**) Comparison of the molecular score with the grade of tubulointerstitial fibrosis and tubular cell damage. Dotted lines represent the threshold values corresponding to histopathology grade 3 (Threshold I) or grade 4 (Threshold II).

The microarray analysis of the discovery set resulted in the identification of a molecular score that reflected the extent of tubulointerstitial fibrosis and tubular cell damage. We explored whether this finding was reproduced in an independent validation set (n = 5; Table 2). The expression levels of HAVCR1, LCN2, SOX, WFDC2, and NKX6-2 were evaluated by microarray analysis (Fig 4A). The results showed coordinated expression of these 5 genes in kidney biopsy specimens, at levels comparable to those observed in the discovery set. The molecular score of each biopsy was calculated using the microarray expression data (Fig 4B). Threshold values corresponding to histopathology grade 3 (Threshold I) or grade 4 (Threshold II) were established using the discovery set results (Fig 4B). Using Threshold I, the predicted grades in all subjects were consistent with the observed histopathology grades (for both tubulointerstitial fibrosis and tubular cell damage, Table 3). When the molecular score was combined with Threshold II, the predicted grades were consistent with the histopathology grades for tubular cell damage, and the predicted grades of 4 subjects matched their tubulointerstitial fibrosis grades. Furthermore, the scatter plots showed a close correlation between the molecular score and the histopathology grade (Fig 4B).

**Table 3:** Prediction of Kidney Histopathology.

	Predicted grade				
	A	B	C	D	E
Molecular score + Threshold I	≥ 3	≤ 2	≤ 2	≥ 3	≥ 3
Molecular score + Threshold II	≥ 4	≤ 3	≤ 3	≥ 4	≥ 4
	Histopathology grade				
	A	B	C	D	E
Tubulointerstitial fibrosis	3–4* ^†^	1* ^†^	2* ^†^	4* ^†^	3*
Tubular cell damage	4* ^†^	1* ^†^	2* ^†^	4* ^†^	5* ^†^

Subjects with a molecular score > Threshold I or > Threshold II were considered to be ≥ 3 or ≥ 4, respectively.

*Histopathology grades were consistent with the predicted value using Threshold I.

^†^Histopathology grades were consistent with the predicted value using Threshold II.

## Discussion

Despite the importance of defining the molecular characteristics of CKD progression, few microarray studies have previously investigated human CKD. In this study, we conducted a microarray analysis of renal biopsies from individuals with CKD to explore their gene expression patterns in relation to relevant histopathological information. This resulted in the identification of gene expression differences that associated with progression of tubulointerstitial fibrosis and tubular cell damage, and these results were validated in a rodent model of CKD. Moreover, the molecular score defined in this study provided a useful marker of the severity of pathological changes.

The present study identified 780 CKD-related differences in gene expression (Fig 1A). The most significantly up-regulated genes were involved in the cell cycle (Fig 1B). This gene cluster included cyclin B2 and cyclin-dependent kinase 1 (CDK1), which are G2/M phase cell cycle regulators. Recent studies reported that the expression levels of cyclin B2 and CDK1 were increased in proliferating tubular epithelial cells from rats with chronic renal failure [3], and that G2/M arrest of proximal tubular epithelial cells was involved in kidney fibrogenesis [12, 13]. Interestingly, our results showed that the expression of CDK1 and cyclin B2 did not correlate with the severity of fibrosis or tubular cell atrophy (Fig 2A), supporting the notion that G2/M arrest in renal cells caused profibrogenic factors to accelerate disease progression in the early phase of CKD.

Hosohata et al. showed that VNN1 could be a biomarker for nephrotoxicant-induced renal injury and the early stage of diabetic nephropathy [14]. In the present study, a gene VNN1 among the CKD-related molecules was also found to be up-regulated in CKD, (8.23 fold increase to control without statistical significance [P > 0.05]). Our data of microarray also showed that the renal expression levels of VNN1 did not correlate with the severity of renal injury, VNN1 would not be an indicator of the progression of renal injury or the therapeutic efficacy.

In this study, a molecular score was calculated from the transcriptional profiles in each biopsy. This score showed a high sensitivity and accuracy for the prediction of tubulointerstitial fibrosis and tubular cell damage (Fig 3). Furthermore, the robustness of the molecular score to predict histological grades was confirmed in the validation set (Fig 4). These findings indicate that the diagnosis of CKD may be improved by using a combination of histology and molecular analyses; this approach may also be valuable for the development of urine or body fluid biomarkers.

Although HAVCR1 (KIM-1) and LCN2 (NGAL) have been characterized as sensitive markers of acute kidney injury [4, 15, 16], recent reports revealed that their kidney expression levels also increased in progressive renal disease [17–20]. The leakage of HAVCR1 and LCN2 into urine could be detected in IgA nephropathy [21]. In the present study, we found increased expression of HAVCR1 and LCN2 in several forms of CKD, and their expression levels were correlated with the degree of tubulointerstitial fibrosis and tubular cell damage. Our findings offer new support for the development of HAVCR1 and LCN2 as biomarkers for prediction of the severity of kidney injury in CKD. Further studies are required in order to explore whether the urinary concentrations of HAVCR1 or LCN2 correlate with the severity of histopathological changes.

This is the first report indicating that WFDC2 expression was altered in CKD. Our study revealed that WFDC2 levels increased with progressive interstitial fibrosis and tubular atrophy in human kidneys. WFDC2 encodes a protein with a highly conserved WAP-domain that is a putative serine protease inhibitor [22]. This is a known biomarker for ovarian carcinoma, and WFDC2 protein was identified in tumor tissue [23], urine [24], and serum [25]. A previous microarray study showed that the kidney expression levels of WFDC2 were increased in renal transplant patients [7]. It was recently shown that WFDC2 expression in myofibroblasts from mouse kidneys mediated renal fibrosis after UUO [25]. The authors reported that WFDC2 suppressed the activity of several proteases, including serine proteases and matrix metalloproteinases, and augmented collagen accumulation in the kidney. Taken together, these findings indicate that WFDC2 could represent a candidate biomarker for fibrosis or tubular injury in the damaged kidney.

We further found that expression of NKX6-2 and SOX9 was increased, and that the levels of these transcripts correlated with the extent of histopathology detected in renal biopsies. These genes encode transcription factors that are related to cell differentiation and development of the brain [26] and the pancreas [27–29]. Interestingly, the expression levels of NKX6-2 and SOX9 were reported to be regulated by the sonic hedgehog (SHH) pathway [30, 31]. Taken together with a previous report indicating that the SHH pathway mediated renal fibrosis in mice after UUO and ischemia-reperfusion injury [32], our results suggested that SHH pathway activation was associated with disease progression in CKD patients, and that SHH inhibition could provide an additional therapeutic approach for progressive renal disease.

In summary, the present study investigated the expression profiles of 53 CKD kidney biopsy specimens using microarrays. The expression levels of HAVCR1, LCN2, WFDC2, SOX9, and NKX6-2 were altered in these patients and correlated with the severity of tubulointerstitial fibrosis and tubular cell damage. These findings provide important information for the development of diagnostic tools and therapeutic agents to predict and prevent progressive renal disease.

## Supporting Information

S1 Checklist (PDF)

S1 Fig Supplemental information. (**A**) Distribution of differences in gene expression between control kidney RNA (Control) and RNA extracted from 31 CKD biopsies with primary glomerular disease or diabetic nephropathy. Microarray analysis was performed and genes down-regulated (z-score < -2.0, green) or up-regulated (z-score > 2.0, blue) in CKD are indicated. (**B**) The numbers of selected genes in the analysis using total biopsies (n = 48) and 31 CKD biopsies were compared. (**C**) Relationship between P values from Kruskal-Wallis test on tubulointerstitial fibrosis and tubular cell damage in the analysis of 31 CKD biopsies. Each symbol represents one gene. Gray or black circles indicate genes with any P values < 0.05; open circles represent genes with both P values > 0.05.
(TIF)
