# Supplementary figures and images for: Molecular Markers of Tubulointerstitial Fibrosis and Tubular Cell Damage in Patients with Chronic Kidney Disease
Source: PLoS One. 2015 Aug 28;10(8):e0136994. doi: 10.1371/journal.pone.0136994 (PMC4552842; doi:10.1371/journal.pone.0136994)

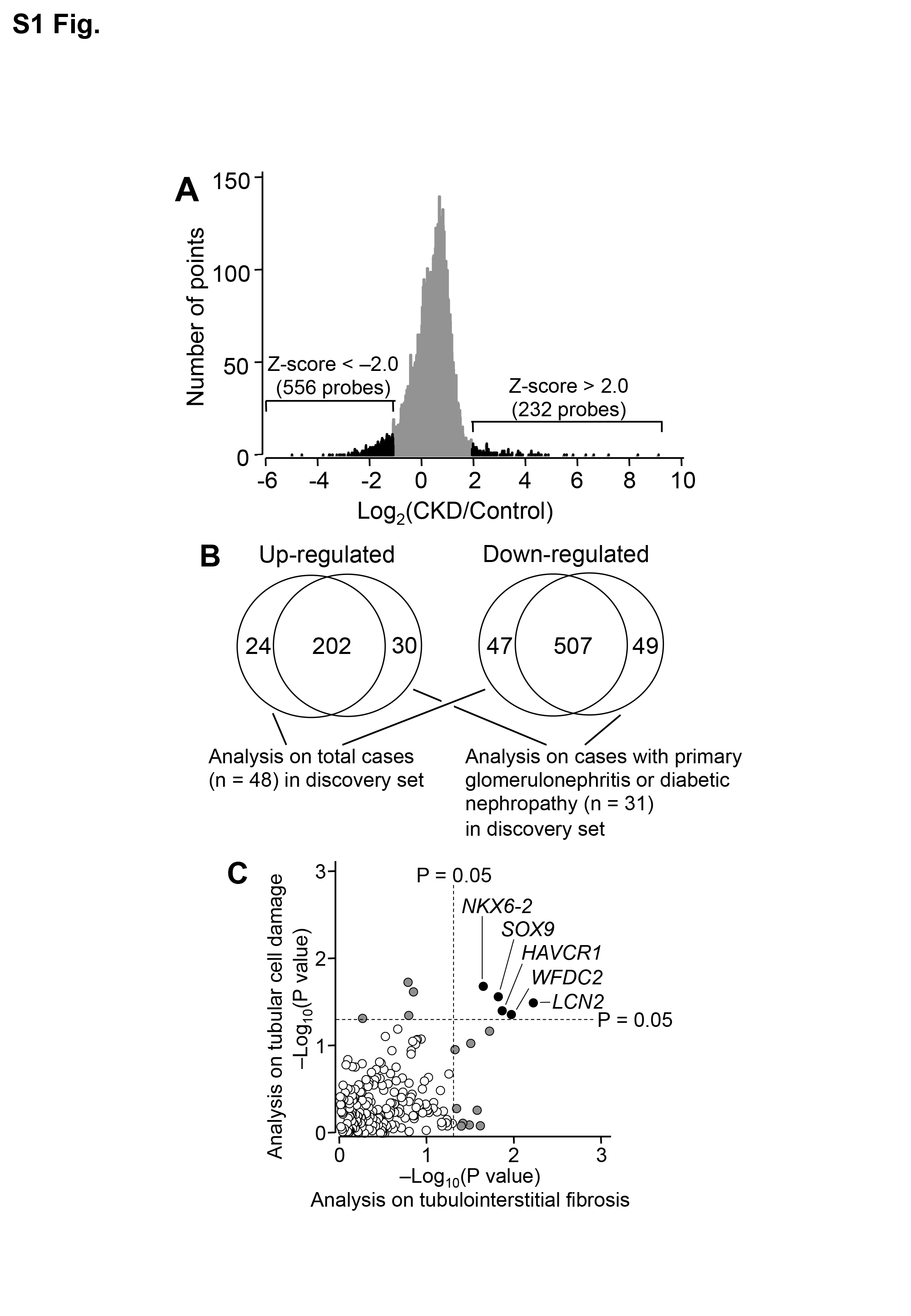

Supplement: S1 Fig — (A) Distribution of differences in gene expression between control kidney RNA (Control) and RNA extracted from 31 CKD biopsies with primary glomerular disease or diabetic nephropathy. Microarray analysis was performed and genes down-regulated (z-score < -2.0, green) or up-regulated (z-score > 2.0, blue) in CKD are indicated. (B) The numbers of selected genes in the analysis using total biopsies (n = 48) and 31 CKD biopsies were compared. (C) Relationship between P values from Kruskal-Wallis test on tubulointerstitial fibrosis and tubular cell damage in the analysis of 31 CKD biopsies. Each symbol represents one gene. Gray or black circles indicate genes with any P values < 0.05; open circles represent genes with both P values > 0.05. (TIF) [file pone.0136994.s002.tif]
